# Disparate volumetric fluid shifts across cerebral tissue compartments with two different anesthetics

**DOI:** 10.1186/s12987-020-00236-x

**Published:** 2021-01-06

**Authors:** Burhan O. Ozturk, Brittany Monte, Sunil Koundal, Feng Dai, Helene Benveniste, Hedok Lee

**Affiliations:** 1 Department of Anesthesiology, Yale School of Medicine, 330 Cedar Street, New Haven, CT USA; 2 Yale Center for Analytical Sciences, Yale School of Public Health, New Haven, CT USA; 3 Department of Biomedical Engineering, Yale School of Medicine, New Haven, CT USA

**Keywords:** Cerebrospinal fluid, Glymphatic transport, Anesthesia, Cerebral compartment volume, Diffusion, Solute transport, Magnetic resonance imaging

## Abstract

**Background:**

Large differences in glymphatic system transport—similar in magnitude to those of the sleep/wake cycle—have been observed during anesthesia with dexmedetomidine supplemented with low dose isoflurane (DEXM-I) in comparison to isoflurane (ISO). However, the biophysical and bioenergetic tissue status underlying glymphatic transport differences between anesthetics remains undefined. To further understand biophysical characteristics underlying these differences we investigated volume status across cerebral tissue compartments, water diffusivity, and T2* values in rats anesthetized with DEXM-I in comparison to ISO.

**Methods:**

Using a crossover study design, a group of 12 Sprague Dawley female rats underwent repetitive magnetic resonance imaging (MRI) under ISO and DEXM-I. Physiological parameters were continuously measured. MRI included a proton density weighted (PDW) scan to investigate cerebrospinal fluid (CSF) and parenchymal volumetric changes, a multigradient echo scan (MGE) to calculate T2* maps as a measure of ‘bioenergetics’, and a diffusion scan to quantify the apparent diffusion coefficient (ADC).

**Results:**

The heart rate was lower with DEXM-I in comparison to ISO, but all other physiological variables were similar across scans and groups. The PDW images revealed a 1% parenchymal volume increase with ISO compared to DEXM-I comprising multiple focal tissue areas scattered across the forebrain. In contrast, with DEXM-I the CSF compartment was enlarged by ~ 6% in comparison to ISO at the level of the basal cisterns and peri-arterial conduits which are main CSF influx routes for glymphatic transport. The T2* maps showed brain-wide increases in T2* in ISO compared to DEXM-I rats. Diffusion-weighted images yielded no significant differences in ADCs across the two anesthesia groups.

**Conclusions:**

We demonstrated CSF volume expansion with DEXM-I (in comparison to ISO) and parenchymal (GM) expansion with ISO (in comparison to DEXM-I), which may explain the differences in glymphatic transport. The T2* changes in ISO are suggestive of an increased bioenergetic state associated with excess cellular firing/bursting when compared to DEXM-I.

## Background

The glymphatic system is a perivascular network for cerebrospinal fluid (CSF) to mix with interstitial fluid (ISF) of the neuropil facilitating brain waste clearance [1]. Solute and fluid transport via the glymphatic system is conceptualized as a dynamic 3-step process: (1) advective driven influx of CSF from the peri-arterial compartment into ISF; (2) CSF-ISF mixing in neuropil driving waste solutes towards peri-venous conduits; and (3) exit of waste to meningeal and extracranial lymphatics for systemic absorption and breakdown [1–3]. The importance of the glymphatic and lymphatic systems for brain health is supported by studies demonstrating glymphatic clearance of amyloid beta (Aβ) [1] and tau [4] and declining waste clearance with aging [5–7]. Furthermore, glymphatic transport is reduced in rodent models of Alzheimer’s disease (AD) [8], cerebral small vessel disease [9–12], and in humans with normal pressure hydrocephalus [13, 14].

The physiological mechanisms controlling glymphatic transport and cross-talk to lymphatic drainage are not fully understood [15–17]. However, several studies have shown that changes in vital physiological states influence glymphatic transport including sleep–wake cycle [18], circadian light–dark cycle [19], vascular pulsatility [11, 20], vasomotion [21], and body posture [22]. Anesthetics also influence glymphatic transport differently, and we previously showed that glymphatic solute transport was 2-fold higher in the rat brain during anesthesia with the alpha2 agonist dexmedetomidine supplemented with low-dose isoflurane (DEXM-I) in comparison to isoflurane only (ISO) [23]. These results were corroborated [24, 25] and further supported in experiments using other alpha-2 agonists such as xylazine mixed with ketamine (KX) which also enhances glymphatic transport in comparison to ISO [25, 26]. Increased slow wave delta and low beta wave power recorded on the electroencephalogram during anesthesia with DEXM-I or KX are associated with more efficient glymphatic system solute and fluid influx when compared to ISO and barbiturates [25]. Notably, an increase in the ISF volume fraction has been recorded in the rodent brain during transition from wakefulness to sleep inferring compartmental volume shifts with slow wave delta activity [18]. In support of the preclinical sleep study [18], a recent MRI study in humans showed increases in CSF compartment volume during sleep compared to wakefulness [27]. The biophysical and bioenergetic tissue status underlying glymphatic transport differences between anesthetics remains undefined. A major goal of the present study was to characterize volume status across tissue compartments, water diffusivity, and T2* values in the rat brain during anesthesia with DEXM-I in comparison to ISO. Based on our previous data documenting increased glymphatic solute transport with DEXM-I compared to ISO [23] we hypothesized that CSF volume would be larger with DEXM-I compared to ISO, and consequently that the parenchymal compartment expands with ISO. We further hypothesized that the ‘apparent diffusion coefficient’, (ADC) representing water diffusion and indirectly the ISF volume fraction [28–31] would be decreased with ISO compared to DEXM-I. We tested our hypotheses using specific MRI sequences which would be sensitive to detect such changes. Proton density weighted (PDW) images were used to quantify volumetric changes of parenchyma and CSF, and diffusion weighted MRI (DWI) was used to quantify the ADC [32]. T2* maps which are representative of neurovascular coupling [33–36] were acquired for further exploration of the bioenergetic state across anesthetics.

## Materials and methods

### Animals

The animal experiments were approved by the local Institutional Animal Care and Use Committee at Yale University (New Haven, Connecticut) and conducted in accordance with the United States Public Health Service’s Policy on Humane Care. Twelve female Sprague–Dawley rats (Charles River Laboratories, Wilmington, MA, USA) between the ages of 12–16 weeks were used. All rats received standard rat chow and water ad libitum and were housed in standard conditions in a 12 h light/dark cycle.

### Experimental design

**Fig. 1:**
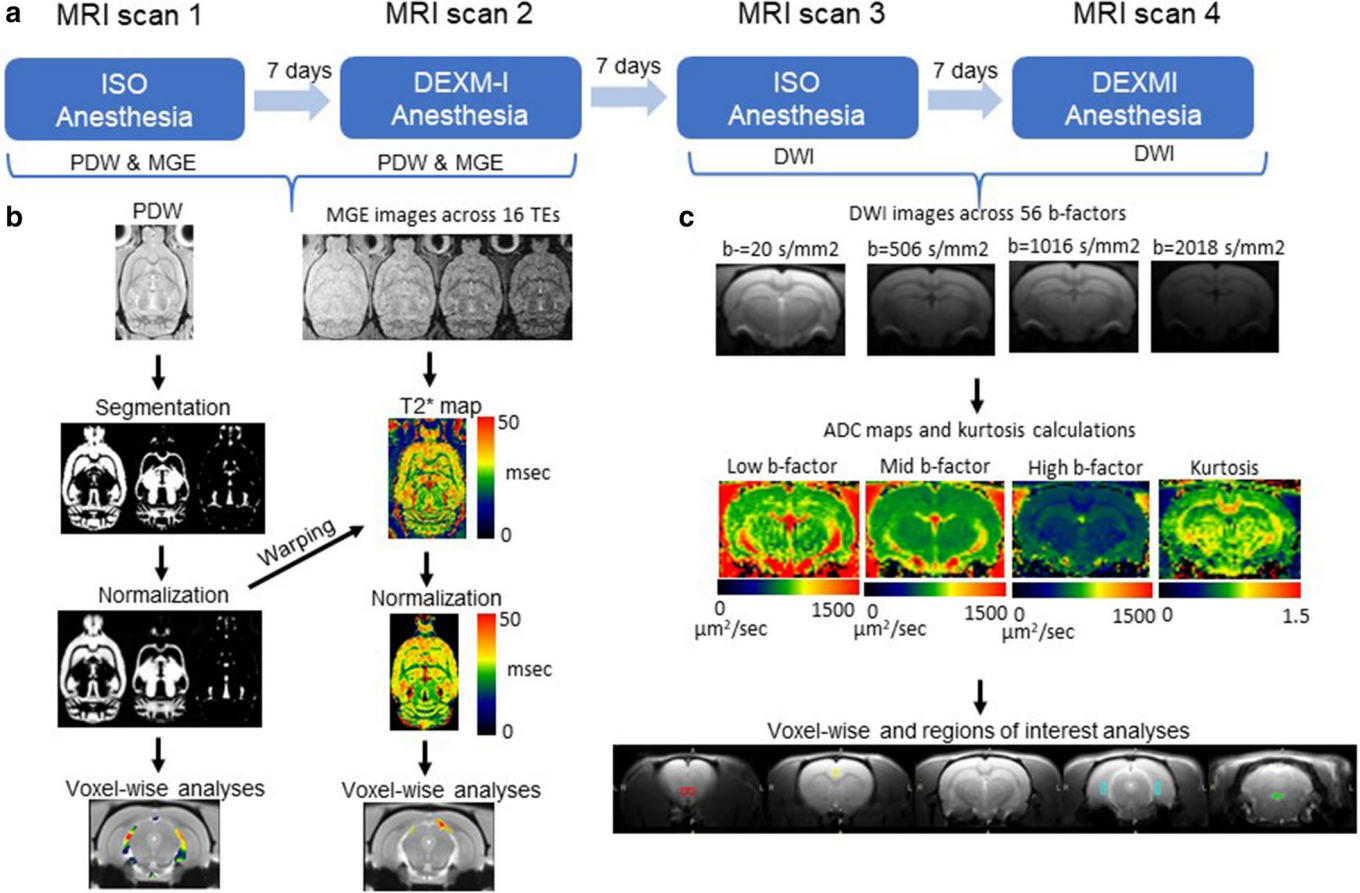
Experimental design of the study and overview of computational analysis. **a** The experiment was designed as cross-over repeated measures study. Twelve rats underwent four MRI scan sessions under two different anesthetic regimens at least 7 days apart to ensure adequate wash-out effects of each anesthetic. **b** Each B1 corrected proton-density weighted (PDW) image was spatially registered and segmented into grey matter (GM), white matter (WM), and CSF using custom made tissue probability maps. Subsequently the images were spatially normalized using deformation fields and processed with voxel-wise analyses. Similarly, each T2* image was spatially normalized using deformation fields derived from PDW images and evaluated by voxel-wise analyses. **c** Apparent diffusion coefficient (ADC) maps were calculated at low-, mid-, and high-evel b-factors in addition to kurtosis maps; and analyzed by both voxel-wise and regions of interest analyses

The experiments to determine differences of ISO and DEXM-I anesthesia on cerebral tissue compartment volumes, T2*, and diffusion were designed as a crossover repeated measures study as shown in Fig. 1. To ensure optimal physiological stability during anesthesia and rapid emergence after scanning, the MRI scan acquisitions were divided into two separate and shorter sessions each lasting ~ 90 min totaling 4 separate MRI scanning sessions. Twelve rats underwent four MRI scan sessions under two different anesthetic regimens at least 7 days apart to ensure adequate wash-out effects of each anesthetic (Fig. 1). The first two scanning sessions measured brain tissue compartment morphometry and bioenergetic changes between DEXM-I and ISO using a 3D PDW and a T2* images, respectively. The last two scan sessions measured the effect of the two anesthetics on the water mobility via ADC maps using a pulsed gradient spin-echo diffusion sequence.

### Anesthesia and preparation for MRI

Anesthesia were induced with 3% isoflurane delivered in a 1:1 Air:O_2_ mixture and after loss of the righting reflex, all rats received hydration maintenance (4 cc/kg 0.9% NaCl) and glycopyrrolate (0.08 mg/kg i.p.). For rats undergoing MRI under ISO, anesthesia was maintained with 2–2.5% isoflurane until the scan was completed. The rats undergoing MRI under the DEXM-I regimen received a bolus of dexmedetomidine (0.007 mg/kg, i.p.) and anesthesia was maintained with a continuous infusion of dexmedetomidine at a rate of 0.009 ± 0.002 mg kg^−1^ h^−1^ administered via a subcutaneous catheter and supplemented with ~ 1% isoflurane delivered with a 1:1 Air:O_2_ mixture, as described previously [37]. The rats were breathing spontaneously throughout the experiments. During each MRI scan session, vital signs including respiratory rate, heart rate and body temperature were measured continuously by non-invasive, MRI compatible monitors (SA Instruments, Stony Brook, NY, USA). Body temperature was kept at 36.5–37.5 °C using a heated waterbed system and oxygen saturation via pulse oximetry was kept at > 96% through the scan sessions. Following completion of MRI scanning, the rats were allowed to recover from anesthesia in their home cage and were observed for ~ 1 h before being returned to the animal facility.

### MRI acquisitions

All MRI acquisitions were performed on a Bruker 9.4T/16 magnet (Bruker BioSpin, USA), controlled by Paravision 6 software. A custom-made volume transmit-receive radiofrequency (RF) coil designed based on Bolinger et al. [38] with an internal diameter of 40 mm was used to acquire anatomical 3D PDW and T2* images. For DWI, a similar designed Bolinger [38] transmit coil with an internal diameter of 50 mm was used for RF excitation and a 20 mm planar surface receive-only RF coil (Bruker BioSpin, USA) was used for RF reception. During MRI, the anesthetized rat was placed in the supine position onto a custom-built 3D-printed animal holder with physiological monitors attached. 3D PDW sequence: 3D PDW images were acquired using a fast low angle shot (FLASH) sequence with the following parameters [39]: repetition time (TR) = 50 ms; echo time (TE) = 4 ms; flip angle (FA) = 7°; number of signal averages (NA) = 4; spatial resolution = 0.23 × 0.23 × 0.23 mm; acquisition time = 27 min. T2* sequence: immediately following the 3D PDW scan, a multi-gradient echo (MGE) sequence with 16 evenly spaced echo times was performed using the following parameters: TR/TE/FA = 60 ms/2 ~ 32 ms/15°; NA = 2; spatial resolution = 0.23 × 0.23 × 0.23 mm; acquisition time = 16 min). To prevent image aliasing artifacts a saturation pulse was applied ventral to the lower jaw in both the PDW and MGE scans. DWI sequence: A 2D, 4 shot echo-planar spin-echo DWI imaging sequence was used for calculation of the ADC using the following parameters: TR/TE = 3000 ms/21 ms; gradient duration = 4 ms; gradient separation = 10 ms; in-plane resolution = 0.3 × 0.3 mm; 11 slices; slice thickness/gap = 2 mm/1 mm; NA = 1; acquisition time = 33 min. A total of 57 b-values ranging from 20 s/mm^2^ to 2518 s/mm^2^ were acquired to calculate the ADC at low, mid and high b-factor [32, 40, 41] ranges. The lowest possible b-factor was 20 s/mm^2^ including the effect of the spoiler gradients. At each b-value, diffusion gradients were applied along three orthogonal directions (read, phase and slice encoding directions). RARE sequence: following the DWI scan, a rapid acquisition with relaxation enhancement (RARE) sequence (TR/TE/FA = 2500 ms/22 ms/90°; in-plane resolution = 0.3 × 0.3 × 2 mm; 11 slices; acquisition time = 1 min), acquired at the same resolution and orientation as the DWI sequence, was performed and utilized as an anatomical template.

### MRI image analyses

#### Morphometry

Voxel-based morphometry (VBM) was implemented using the SPM12 software package (https://www.fil.ion.ucl.ac.uk/spm) described previously [39, 42]. PDW images were first corrected for B1 inhomogeneity, followed by spatial registration and tissue segmentation into three tissue compartments: grey matter (GM), white matter (WM), and CSF. Spatial registration was performed using DARTEL image processing pipelines and custom-made tissue probability maps [10, 42], and the spatially normalized images were subsequently smoothed by a Gaussian smoothing kernel of 0.6 mm. Voxel-wise statistical analysis was performed to identify local morphological differences using a paired t-test and statistical significance was reported at p < 0.05 after correcting for multiple comparisons via a false discovery rate (FDR) algorithm [43].

#### T2* analysis

T2* differences between the two anesthetic groups were also characterized by voxel-wise analysis [39]. 3D T2* maps were first calculated from the 3D MGE image intensities by assuming mono-exponential signal decay between the detected signals and the echo times using the following formula: *S(TE)* = *S*_*0*_*e*^−TE/T2*^, where S_0_, TE, and T2* represent the PDW signal, echo time and the transverse relaxation time, respectively. Each 3D T2* map was then spatially normalized by applying the deformation fields derived from the PDW image analysis and smoothed with an isotropic Gaussian kernel of 0.6 mm. Mean compartmental T2* values were calculated between 0.1 and 99% percentile to exclude any possible outliers that may skew the mean. A voxel-wise statistical analysis was subsequently performed to identify T2* differences using a paired t-test and statistical significance was reported at p < 0.05 after correcting for multiple comparisons via FDR [43].

#### Diffusion analysis

DWI images acquired along three orthogonal gradient encoding directions were combined by taking the geometric means of signal intensities for each b-factor. DWI images were then motion corrected using a rigid alignment algorithm in SPM12 which realigns each individual DWI image with the mean of all the DWI images. ADC maps were calculated separately for three different ranges of b-factors: low-range (*b* = 20–205 s/mm^2^), mid-range (*b* = 235–1016 s/mm^2^), and high-range (*b* = 1117–2518 s/mm^2^) using the standard Stejskal and Tanner equation [44]: $S(b) = S_{0} e^{ - b*ADC}$, where $S_{0}$, $b$, and $ADC$ represent PDW signal, b-factor, and ADC, respectively. DWI image intensities, $S(b)$, were log transformed and fitted as a function of the b-factors using an unweighted linear least square fit algorithm to calculate each ADC map. Although the assumption of a mono-exponential signal decay is a standard approach for calculating ADC using the Stejskal and Tanner equation, DWI signals are known to deviate from the mono-exponential signal decay at very low (below *b* = 205 s/mm^2^) and high b-factors (above *b* = 1200 s/mm^2^). Therefore, a biexponential function is used instead to model the DWI signals across a wider range of b-factors (20–2518 s/mm^2^) and is expressed as follows [32]:

1 $$\frac{S(b)}{{S{}_{0}}} = f_{IVIM} e^{{ - bD^{*} }} + (1 - f_{IVIM} )e^{{ - bADC_{0} + (bADC_{0} )^{2} K/6}} ,$$

where $S{}_{0}$, $f_{IVIM}$, $D^{*}$, $ADC_{0}$, and $K$ are the PDW signal, fraction of intravoxel incoherent motion, virtual diffusion coefficient, ADC, and kurtosis, respectively. Kurtosis analysis can be regarded as an extension of the conventional ADC analysis and provides a means to also consider non-Gaussian diffusion of water [45, 46]. Note that when K = 0, the ADC reflects a pure Gaussian diffusion condition. Since $D^{*}$ is reported to be very high and $f_{IVIM} < < 1$, Eq. (1) can be simplified when the b-factor is above *b* = 235 s/mm^2^ and the first term is negligibly small compared to the second term expressed as:

2 $$S(b) = S{}_{0}e^{{ - bADC_{0} + (bADC_{0} )^{2} K/6}} .$$

leaving three unknown variables. The unknown variables were calculated by using the Levenberg–Marquardt non-linear least square algorithm using an in-house code written in MATLAB (MathWorks, Natick, MA, USA). Initial conditions of $ADC_{0}$ and $S{}_{0}$ were estimated from the results of mid b-factor ADC, $S_{0\_mid}$ and $ADC_{mid}$, using the standard Stejskal and Tanner equation and $K$ was set to 0.623 as reported previously [41]. $S{}_{0}$, $ADC_{0}$, and $K$ were further constrained within the range of 0.5*$S_{0\_mid}$ < $S_{0}$ < 2.0 $S_{0\_mid}$, 0.5*$ADC_{mid}$ < $ADC_{0}$ < 2.0 $ADC_{mid}$ and 0 < $K$ <  2.0, respectively.

ADC maps at low-, mid-, and high b-factors and $K$ maps were calculated for each session as shown in Fig. 1c, and aligned rigidly between scans followed by 6 mm Gausssian smoothing to accommodate voxel-wise analyses. Voxel-wise statistical analyses of the ADC maps were performed using a paired t-test and statistical significance was reported at p < 0.05 after correcting for multiple comparisons via FDR [43]. In addition, the regions of interest were drawn onto the 2D RARE T2W images using ITK SNAP [47] (http://www.itksnap.org/pmwiki/pmwiki.php) and superimposed onto both ADC and kurtosis maps to extract the means. ROI analysis of ADCs across anesthetic groups focused on tissue areas where morphometric differences were detected via the VBM analysis.

### Statistical analyses

Sample size for the crossover study design using MRI based morphometry between the two anesthetics was determined based on previous experience [39]. A linear mixed-model with a heterogeneous variance covariance matrix for repeated measures over time was used to analyze the impact of the multiple scan sessions and type of anesthetic on each physiological parameter and body weight within the same rat with fixed effects of ‘time’ (MRI sequence), group (anesthetic) and the interaction between time and group. Group differences were calculated using a post-hoc pairwise Fisher’s least significant difference (LSD) that did not adjust for multiple comparisons. To evaluate group differences (DEXM-I versus ISO) between the total volume of the tissue compartments [CSF, WM + GM, total intracranial volume (TIV)] a two-tailed paired t-test was used. For the regional ADC analysis, we used a linear mixed-models with fixed effects of ‘time’ (b-factor ranges), group (anesthetics) and the interaction between b-factor range and group. Group and b-factor dependent ADC differences were assessed using a post-hoc pairwise post-hoc pairwise Fisher’s least significant difference (LSD) that did not adjust for multiple comparisons. For comparison of mean T2* values of the tissue compartments (CSF, WM + GM) a two-tailed paired t-test was used. In addition, the regional differences were corroborated with Statistical Parametric Mapping using the SPM12 (http://www.fil.ion.ucl.ac.uk/spm) software package. For all statistical analyses, p < 0.05 was considered to be significant. All statistical analyses were performed using XLSTAT Software (Version 2016.5, Addinsoft, Paris France) and SPSS (IBM SPSS Statistics, version 26).

## Results

### Physiological data

We used a linear mixed-model for repeated measures to analyze the impact of the multiple scan sessions (time) and type of anesthetic (group) on each physiological parameter and body weights for the same rat with fixed effects of ‘time’, group and the interaction between time and group. Table 1 summarizes the physiological parameters and body weights of the rats across the scans and anesthetics. Respiratory rate and temperature did not change between scan sessions and did not differ across the type of anesthetics (Table 1). Similarly, body weights measured before each scan was also stable over the course of the study conducted over ~ 1 month (Table 1). As expected, heart rate differed by type of anesthetics due to the sympatholytic effects of the alpha-2 agonist dexmedetomidine (Table 1).

**Table 1:** Summary of statistical vital signs analysis across anesthetics

		DEXM-I (N = 12)		ISO (N = 12)						
Parameter	Scan sequence	Mean^a^	SE	Mean^a^	SE	Difference^b^	SE	P value	L95%	U95%
Heart rate (beat/min)	PDW + MGE	276.4	3.9	361.9	11.4	− 85.550	12.098	**0.000**	− 111.574	− 59.526
	DWI	280.8	7.1	352.8	10.3	− 71.908	12.516	**0.000**	− 98.052	− 45.765
Respiratory rate (breath/min)	PDW + MGE	51.0	1.5	52.6	0.82	− 1.600	1.677	0.353	− 5.133	1.933
	DWI	50.7	1.3	51.7	1.1	− 1.050	1.695	0.542	− 4.569	2.469
temperature (°C)	PDW + MGE	37.03	0.04	37.08	0.08	− 0.050	0.086	0.571	− 0.234	0.134
	DWI	36.97	0.04	37.09	0.04	− 0.125	0.055	**0.033**	− 0.239	− 0.011
Body weight (g)	PDW + MGE	285.5	10.9	284.4	9.8	1.083	14.658	0.942	− 29.334	31.501
	DWI	290.6	5.5	283.5	3.9	7.083	6.790	0.309	− 7.087	21.253

^a^Data are presented as least square means and SE’s

^b^Least square mean differences compare DEXM-I vs. ISO groups for each scan sequence (PDW + MGE and DWI)

### Morphometry

**Fig. 2:**
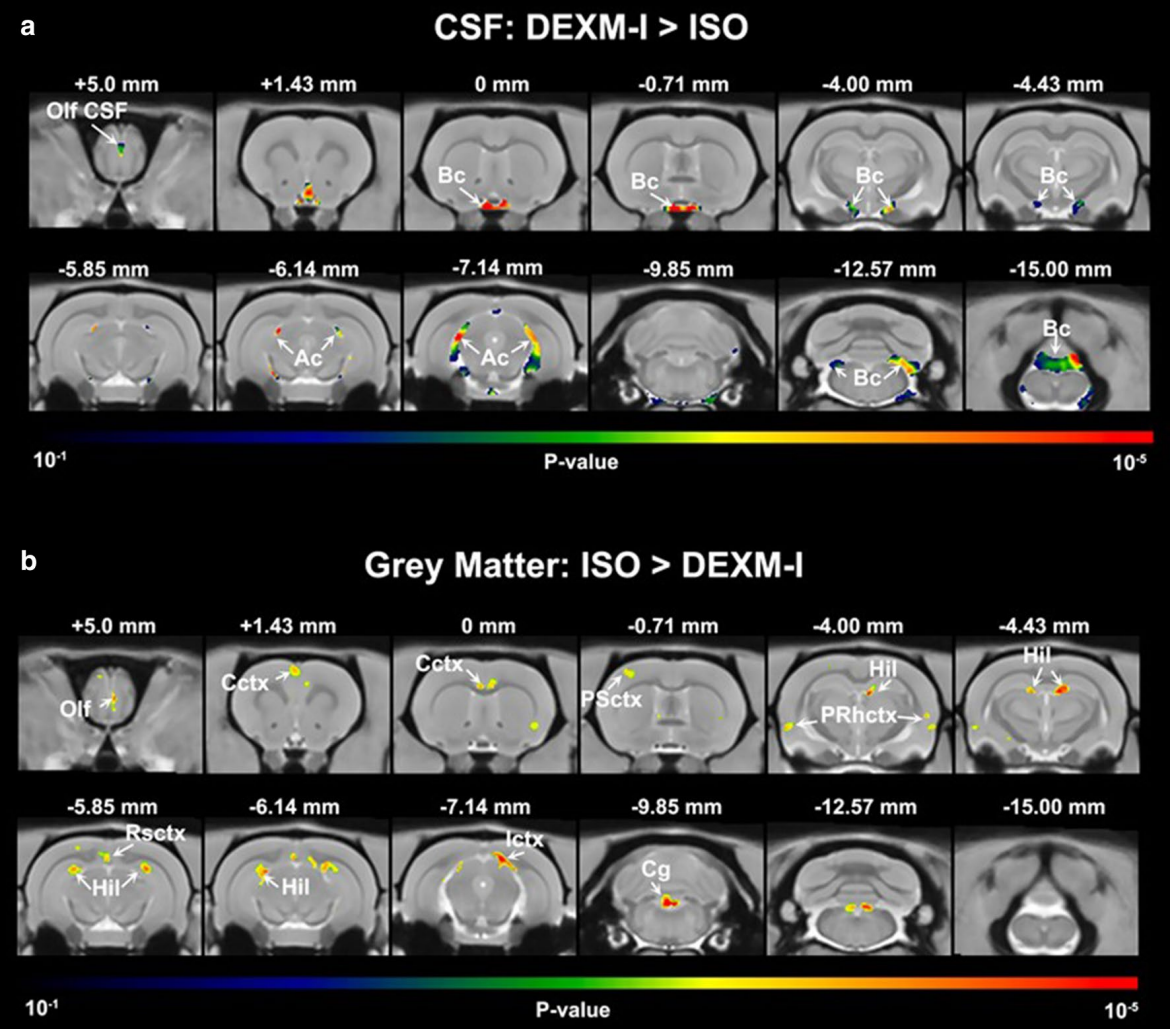
Voxel based analysis of brain tissue compartment across ISO and DEXM-I. **a** Statistical parametric maps (color coded for p-values) were calculated, corrected for FDR < 0.05 and overlaid onto population averaged PDW images to display anatomical areas with significantly enlarged CSF volume in DEXM-I compared to ISO anesthetized rats. The anatomical levels in relation to Bregma is listed above each frame. **b** Statistical parametric maps to display anatomical areas with significantly lower voxel volumes in grey matter in DEXM-I compared to ISO anesthetized rats. The anatomical levels in relation to Bregma is listed above each frame. *Olf CSF* Olfactory bulb associated CSF, *Bc* basal cistern, *Ac* ambient cistern, *Cctx* cingulate cortex, *PSctx* parietal (somatosensory) cortex, *PRhctx* peri-rhinal cortex, *Hil* hilus, dentate gyrus, *Ictx* insular cortex, *Cg* central grey

**Fig. 3:**
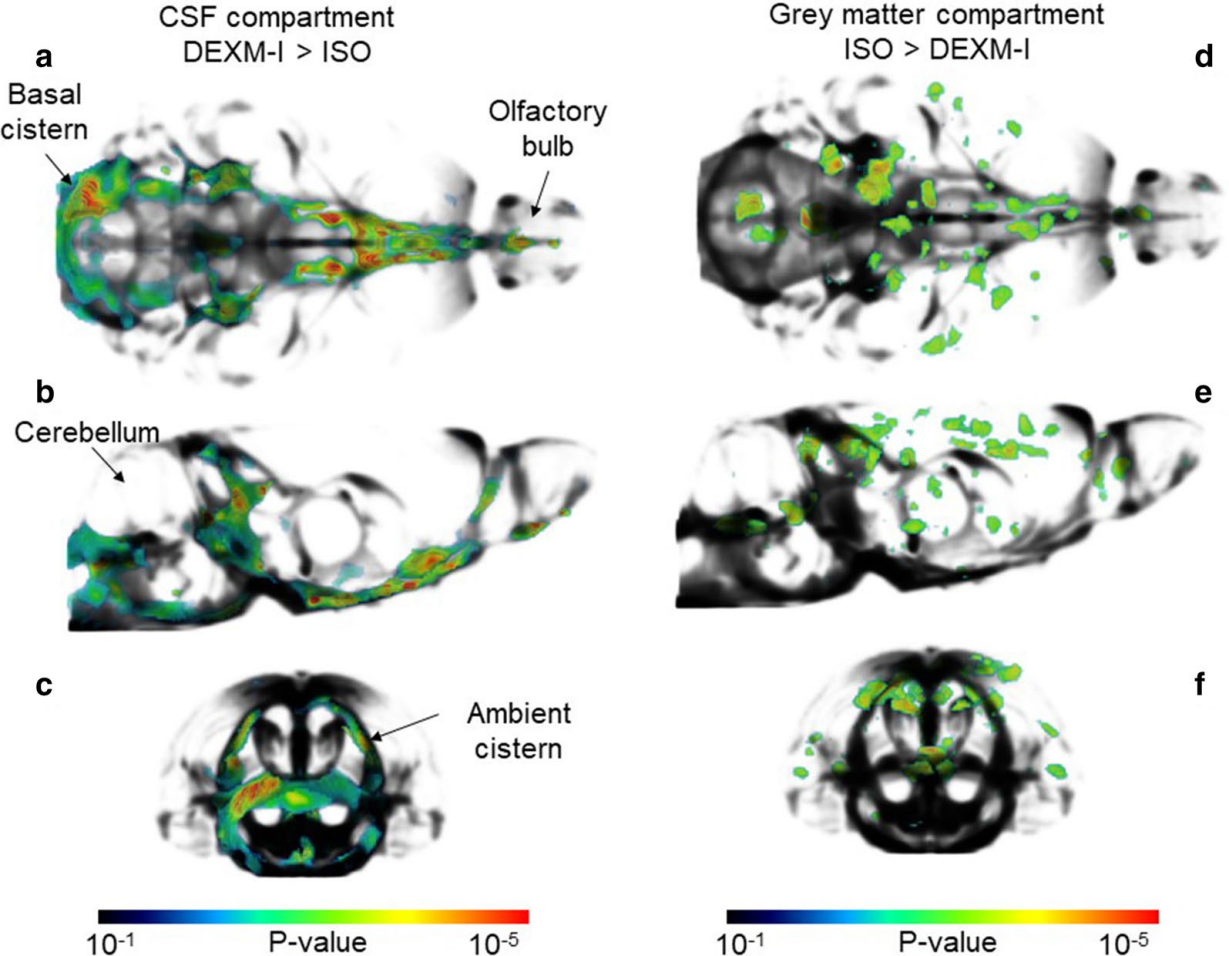
Visualization of CSF and tissue volume enlargements across ISO and DEXM-I rats. **a**–**c** 3D volume rendered statistical parametric maps (color-coded for p-values) are overlaid onto 3D volume rendered CSF binary map (black) to illustrate the CSF volume enlargements in DEXM-I in comparison to ISO. **a** Ventral view showing the basal cistern and peri-arterial conduits along the circle of Willis. **b** Side view illustrating the CSF volume enlargement at the level of the basal and ambient cisterns. **c** Caudal view to show the CSF enlargement related to the ambient cistern. **d–f** 3D volume rendered statistical parametric maps (color-coded for p-values) shown in three orthogonal view overlaid onto 3D volume rendered CSF map (black). The statistically significant areas of tissue expansion are shown to be scattered across the cortex. Few of the enlarged focal areas are located in proximity to CSF spaces including the aqueduct and ambient cistern

Following image segmentation using the tissue probability maps; parenchymal, CSF, and total intracranial volumes were calculated. There was no significant difference in the total intracranial volume between the two anesthetic groups (DEXM-I (N = 12) 2015 ± 86 mm^3^ versus ISO (N = 12) 2021 ± 95 mm^3^, p = 0.340). However, the DEXM-I group displayed a ~ 6% higher CSF compartment volume when compared to the ISO group (DEXM-I (N = 12) 190 ± 20 mm^3^ versus ISO (N = 12) 180 ± 19 mm^3^; p < 0.001), whereas the ISO group yielded a 1% higher parenchymal volume (ISO (N = 12) 1841 ± 90 mm^3^ versus DEXM-I (N = 12) 1826 ± 81 mm^3^; p = 0.024) when compared to the DEXM-I group. To spatially localize morphometric volume differences between DEXM-I and ISO groups, a voxel-wise VBM analysis was performed. As shown in Fig. 2, 2D parametric maps of color-coded p-values from the paired-tests were overlaid onto the corresponding population averaged PDW anatomical maps to display areas with significant differences. In agreement with the quantitative volume data, the voxel-wise analysis revealed a statistically significantly larger CSF volume in DEXM-I compared to ISO groups localized to the basal and ambient cisterns, and the olfactory subarachnoid space (Fig. 2a). Further, enlarged peri-vascular CSF conduits were noted along arteries of the circle of Willis, longitudinal hippocampal artery, and the anterior cerebral artery and its branches (the medial orbitofrontal artery and the lateral orbitofrontal artery). Anatomical visualization of statistically significant CSF compartment enlargements in DEXM-I in comparison to ISO was also captured via 3D volume rendered color coded p-value maps overlaid onto the CSF compartment binary template displayed in black (Fig. 3a–c). From the 3D displays it is evident that the CSF compartment enlargements in DEXM-I (in comparison to ISO) are greatest in basal cisterns and peri-arterial space associated with the circle of Willis and large arteries feeding the forebrain and the hippocampus (Fig. 3a–c).

The voxel-wise analysis of significantly larger parenchymal (GM) tissue volume in ISO compared to DEXM-I anesthetized rats is shown in Figs. 2b, 3d–f. The scattered distribution of multiple areas significant for parenchymal grey matter ‘swelling’ in ISO compared to DEXM-I is striking and involve the olfactory bulb, dentate gyrus (hilus region), primary somatosensory, insular, visual, auditory, cingulate, perirhinal and retrosplenial cortices as shown in Figs. 2b, 3d–f. Notably, some of the GM areas with local tissue ‘swelling’ were observed in close proximity to CSF spaces (Fig. 3d–f). The VBM analysis revealed no volume differences in white matter regions between DEXM-I and ISO.

### Apparent diffusion coefficients and kurtosis

We first conducted a voxel-wide analysis of ADC differences in the brain for low-, mid-, and high-b-factor ranges across DEXM-I and ISO anesthetized rats. There were no tissue voxels that survived the statistical analysis with FDR correction neither for DEXM-I ADC > ISO ADC nor ISO ADC > DEXM-I ADC regardless of b-factor range. To further validate the voxel-wise analysis we also performed a ROI based analysis. Specifically, we selected ROIs guided by the VBM analysis where parenchymal enlargement was evident including the olfactory bulb, cingulate cortex, ventral hippocampus (Table 2). First, the mean ADCs for low-, mid- and high b-factors, were ~ 800 μm^2^/s, ~ 600 μm^2^/s, and ~ 500 μm^2^/s, respectively, in brain tissue. The significant and decreasing trend in b-range dependent ADC values is expected and well-documented as the brain tissue DWI signal loss is known to deviate from the assumed single mono-exponential decay at low and high b-factors in particular. This phenomenon can be demonstrated by measuring ADCs using the same b-factor ranges in a dimethyl sulfoxide (DMSO) phantom. Additional file 1: Fig. S1 compares the fitting of DWI signals between a DMSO phantom and brain tissue and clearly shows a poor linear fit in vivo. ADC of the DMSO phantom was ~ 680 μm^2^/s which is comparable to that of brain tissue ADCs, independent of b-factor ranges, as DWI signals in the DMSO phantom retain a mono-exponential signal decay over all b-factor ranges. In brain tissue, however, the slope of the DWI signal becomes steeper than that of the DMSO phantom at the low b-factor range which is attributed to perfusion related intra-voxel incoherent motion [32]. In the mid b-factor range, the slope of tissue DWI signal follows a mono-exponential trend similar to the DWI signals of the DMSO phantom. At the high b-factor range, the slope of the tissue DWI signals decrease in comparison to DMSO DWI signals due to the so-called non-Gaussian diffusion kurtosis effect [46, 48, 49]. To further study the relationship between kurtosis and ADC calculated at high b-factor, DWI signals were simulated using Eq. (1) using the parameters from a previous study [41] and decreased kurtosis by 20% and 40% as shown in Additional file 2: Fig. S2. Reduction in kurtosis by 20% and 40% yielded 9% and 17% increase in ADCs, respectively. Table 2 shows that for the mid- and high-level b-factor ADCs, there were no statistical regional differences across anesthesia groups in agreement with the voxel-based analysis. For the olfactory bulb a statistically significant increase in the low b-factor ADC only was observed with ISO compared to DEXM-I which may be contributed to perfusion related intra-voxel incoherent motion in this region (Table 2). As for kurtosis, Table 3 summarizes kurtosis values across the same four ROIs showing heterogeneity across brain regions (0.6–0.9) similar to values reported in previous studies [41, 50, 51]. Kurtosis values extracted from each ROIs did not differ across the DEXM-I and ISO groups (Table 3).

**Table 2:** Statistical analysis of ADCs for brain regions across the two anesthetic groups

		DEXM-I (N = 12)		ISO (N = 12)						
Brain region	ADC µm^2^/s	Mean^a^	SE	Mean^a^	SE	Difference^b^	SE	P value	L95%	U95%
Olfactory bulb	Low ADC	720.7	37.3	848.1	37.3	− 127.450	52.834	**0.025**	− 237.021	− 17.879
	Mid ADC	597.7	12.0	612.5	12.0	− 14.708	16.991	0.396	− 49.945	20.528
	High ADC	420.6	8.8	431.6	8.8	− 11.092	12.490	0.384	− 36.994	14.810
Somatosensory cortex	Low ADC	761.6	10.0	769.7	10.0	− 8.167	14.161	0.570	− 37.534	21.201
	Mid ADC	655.7	4.6	663.7	4.6	− 8.000	6.472	0.229	− 21.421	5.421
	High ADC	509.7	3.1	510.9	3.1	− 1.167	4.424	0.794	− 10.341	8.008
Cingulate cortex	Low ADC	768.4	19.6	782.6	19.6	− 14.175	27.747	0.615	− 71.718	43.368
	Mid ADC	667.2	6.6	663.8	6.6	3.383	9.358	0.721	− 16.024	22.791
	High ADC	516.7	4.5	523.8	4.5	− 7.158	6.335	0.271	− 20.297	5.980
Ventral hippocampus	Low ADC	830.8	24.0	857.3	24.0	− 26.467	33.882	0.262	− 96.734	43.801
	Mid ADC	702.4	9.9	699.7	9.9	2.708	14.063	0.849	− 26.456	31.873
	High ADC	528.0	7.0	539.4	7.0	− 11.4	9.896	0.262	− 31.922	9.122

^a^Data are presented as least square means and SE’s

^b^Least square mean differences compare DEXM-I vs. ISO groups for each ADC extracted from low-, mid- and high b-factor ranges

**Table 3:** Statistical analysis of kurtosis values for brain regions across the two anesthetic groups

	Kurtosis DEXM-I (N = 12)		Kurtosis ISO (N = 12)						
Brain region	Mean^a^	SE	Mean^a^	SE	Difference^b^	SE	P value	L95%	U95%
Olfactory bulb	0.908	0.021	0.908	0.021	− 0.001	0.030	0.985	− 3.046	3.045
Cingulate cortex	0.699	0.021	0.656	0.021	0.043	0.030	0.158	− 3.003	3.088
Somatosensory cortex	0.730	0.021	0.699	0.021	0.031	0.030	0.307	− 0.029	0.091
Ventral hippocampus	0.681	0.021	0.639	0.021	0.043	0.030	0.160	− 3.003	3.088

^a^Data are presented as least square means and SE’s

^b^Least square mean differences compare DEXM-I vs. ISO groups

### T2* maps signal values

**Fig. 4:**
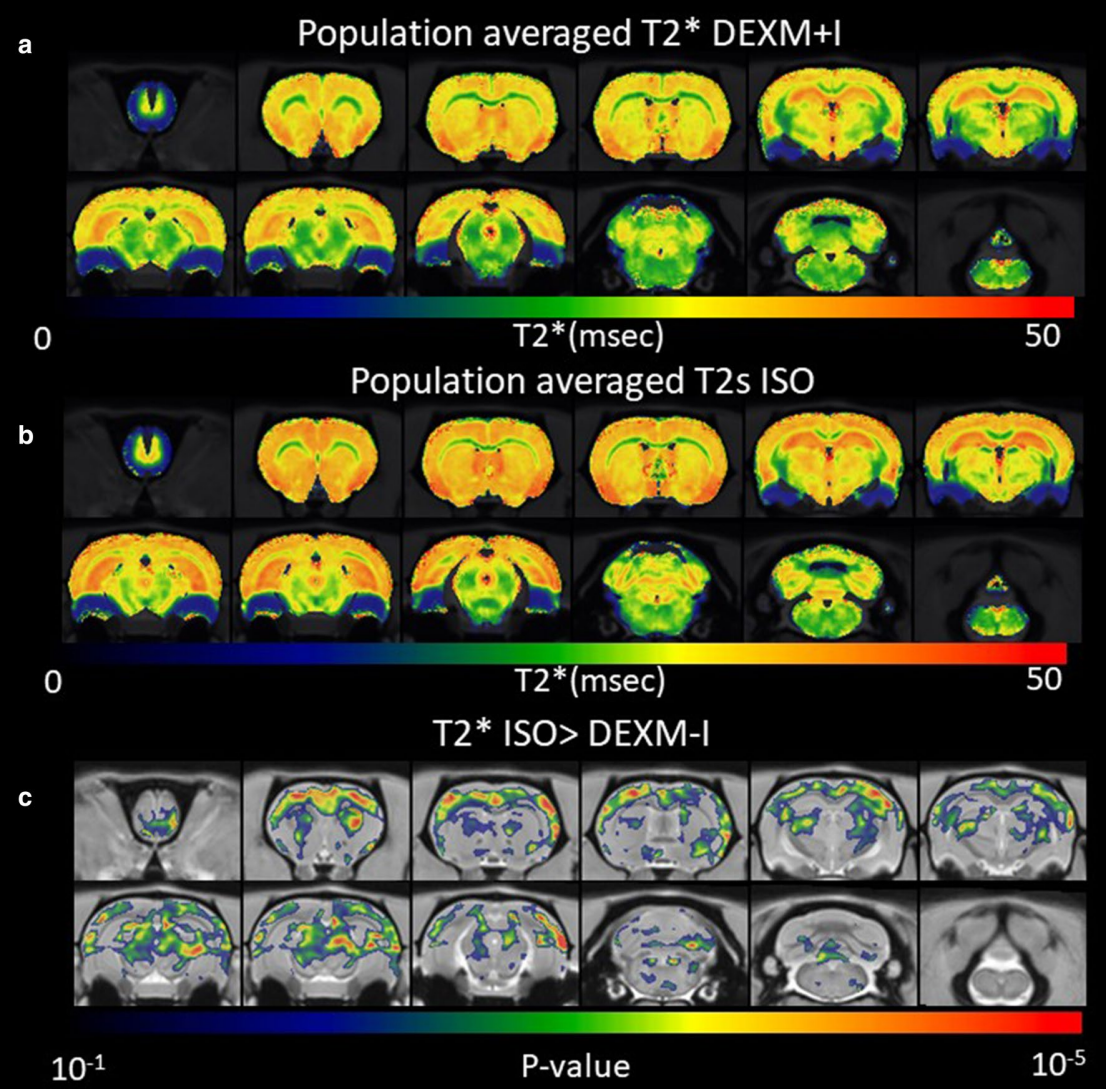
T2* is increased in the brain during anesthesia with ISO compared to DEXM-I. Voxel-wise T2* results between the ISO and DEXM-I group. **a** Spatially normalized population averaged T2* maps of DEXM-I group. **b** Spatially normalized population averaged T2* maps of ISO group. **c** Statistical parametric maps (color coded for p-values) were calculated, corrected for FDR < 0.05 and overlaid onto population averaged PDW MRI images to display anatomical areas with significantly higher T2* in ISO group in comparison to the DEXM-I group

Population averaged color-coded T2* maps are shown for the DEXM-I and ISO groups in Fig. 4a, b, respectively. In both groups, T2* contrast enables anatomical distinction between GM and WM tissue compartments since myelin in WM is known to exhibit lower T2* than GM**.** The mean T2* values were significantly higher in the ISO anesthetized rats compared to DEXM-I rats (ISO = 30.6 ± 1 ms vs. DEXM-I = 28.8 ± 1.1 ms; p < 0.001). The voxel-wise, Statistical Parametric Mapping (SPM) analysis for the condition T2* ISO > T2* DEXM-I revealed widespread higher T2* values in cortical as well as subcortical regions, corroborating the quantitative mean T2* analysis between the two anesthesia group. No T2* differences were found in the voxel-wise SPM analysis for the condition T2* DEXM-I > ISO (Additional file 3: Additional reference).

## Discussion

The most profound changes in glymphatic system fluid and solute transport are observed during transition from wakefulness to deep sleep [18] or across different anesthesia regimens [23–26, 52]. Specifically, in mice, glymphatic transport decreased by ~ 95% upon arousal from deep sleep [18], and we previously documented 2-fold higher glymphatic solute transport during anesthesia with DEXM-I compared to ISO [23]. We hypothesized that the increased glymphatic transport with DEXM-I in comparison to ISO would be associated with compartmental volume changes. The result of the PDW VBM data analysis corroborated our hypothesis demonstrating that the total CSF volume was significantly increased with DEXM-I compared to ISO. Visualization of the CSF volume changes revealed expansions in DEXM-I (compared to ISO) at the level of the basal and ambient cisterns and large peri-arterial space conduits along the ventral surface of the brain (Figs. 2, 3). These exact same conduits function as the main CSF influx routes for glymphatic transport [22, 53], and their enlargements may in part explain the increased fluid and solute transport observed with DEXM-I over ISO [17, 23]. Higher CSF volume flow would enhance influx via penetrating cortical arteries thereby increasing glymphatic CSF-ISF exchange and ultimately waste drainage. The physiological explanation for the CSF expansion in DEXM-I over ISO may be explained by the direct vasoconstrictive effect of DEXM which has been documented in humans [54–56] and rats [23]. Thus, alpha‐2 adrenergic agonists mediate sympatholytic effects through activation of centrally and peripherally located alpha‐2 adrenoceptors [54, 55]. On the other hand, the direct vasodilatory effect of ISO [57] may have exaggerated these changes when compared to dexmedetomidine. It is important to note that the respiratory rate across the two anesthesia groups was within similar ranges (Table 1) and it is unlikely therefore that differences in arterial pCO_2_ contributed to vasoconstriction or vasodilation across groups. In our previous work using the exact same anesthetic regimens we documented that arterial blood gas values including pCO_2_ were similar across groups [23]. It is possible, however, that CSF production might have been different across DEXM-I and ISO conditions but static measures of tissue compartments by the VBM analysis cannot address this possibility.

In accordance with the Monroe-Kellie doctrine for normal brain and normal intracranial pressure, we also demonstrated that the parenchymal volume was significantly increased (~ 1%) in ISO compared to DEXM-I. Specifically, the VBM tissue analysis with ‘ISO > DEXM-I’ revealed multiple focal ‘swollen’ areas which appeared to be scattered throughout the forebrain (Fig. 3). The focal enlarged areas with ISO were distributed mainly in the cortices across both hemispheres as well as in the hippocampus. Further, a smaller fraction of the ‘swollen’ tissue regions was observed near CSF reservoirs (Fig. 3). The scattered areas of parenchymal enlargement in the ISO group might be interpreted as cellular ‘swelling’, or they could represent changes in the cerebral vascular volume. If the focal parenchymal enlargements represent areas of cellular swelling, this will infer an associated ISF volume fraction reduction, increased hydraulic resistance and decreased glymphatic solute and fluid transport with ISO in comparison to DEXM-I. Alternatively, a fraction of the volumetric tissue ‘enlargements’ in the forebrain with ISO compared to DEXM-I positioned close to CSF spaces might be ‘misrepresented’ CSF voxels due to volume averaging effects of the PDW images. This may have led some voxels to appear more “GM” in ISO compared to DEXM-I. However, for ‘expanded’ tissue areas in deeper portions of cortex e.g. parietal or cingulate cortex as shown in Fig. 2b this argument does not hold.

Changes of the ISF volume fraction are sensitive to detection by diffusion imaging via the ADC and/or kurtosis [58–60]. The ADC has been shown to decrease rapidly with acute ischemia [58, 61], in tissue with acute cytotoxic edema [31, 62] and with neuronal activation [63, 64]. Acute cerebral ischemia and cortical spreading depression are associated with ~ 40–50% reduction of the ISF volume fraction as measured by the tetramethylammonium technique [65–70]. We expected to observe decreases in the ADC and increases in kurtosis values in areas of parenchymal enlargement (inferring ISF reduction) with ISO compared to DEXM-I. Surprisingly, our analysis of ADC and kurtosis values did not reveal global or focal differences across the two anesthesia groups. While the positive findings of tissue compartment enlargement in ISO compared to DEXM-I is supportive of differences in cellular volume and the ISF volume fraction, the ADC and kurtosis measures did not confirm this finding. The discrepancy may be explained as follows: (1) smaller areas of ‘parenchymal swelling’ with ISO compared to DEXM-I are not associated with cellular swelling and ISF changes but may be caused by enlargement of the vascular compartment; (2) the previously reported dramatic (~ 50%) ISF volume fraction enlargement reported in somatosensory cortex during deep sleep compared to wakefulness [18], may be more subtle compared to the volume changes across the two short-duration (90-min) anesthesia and diffusivity/ADC may therefore not be sufficiently sensitive to detect these changes; or (3) the 2D ADC/kurtosis sequence used in our study had insufficient spatial resolution to capture small changes in cellular volume and ISF volume.

The mechanisms underlying the increase in the ISF volume fraction during deep sleep compared to wakefulness has been attributed to decreases in central norepinephrine (NE) activity associated with slow-wave delta oscillations [18]. Intriguingly, it has also been revealed that astrocytes regulate the sleep–wake cycle, (in response to norepinephrine) by modulating the levels of extracellular ions which in turn drive neuronal responsiveness [71]. The ISF volume expansion during deep sleep induced by ionic shifts is thought to reduce the hydraulic restriction for solute and fluid transport through neuropil [18, 71], and improve glymphatic transport. In the same vein, glymphatic system transport increases with alpha2 agonists such as dexmedetomidine and KX in comparison to ISO have also been attributed to their ability to decrease NE and increase the power of slow wave delta oscillations [23]. This is because alpha-2 agonists such as DEXM inhibits NE release at the level of the locus coeruleus (LC), which is the main source of NE in the CNS. However, we acknowledge that that because the DEXM-I regimen in our study includes ISO at a lower concentration than the ISO only condition our results cannot distinguish between effects of dexmedetomidine, effects of isoflurane at ~ 1%, or effects of the combination when compared to high dose Isoflurane. Evidence for ISF changes related to changes in central NE activity in vivo is scarce and to our knowledge no study has applied diffusion MRI for comparison of DEXM-I and ISO. A diffusion MRI study in mice comparing ISO and wakefulness reported no differences in ADCs between the two arousal states, however, ISO does not inhibit NE activity limiting the overall interpretation in relation to NE activity [52]. Our previous study in humans comparing sleep/awake state revealed an increase in the CSF compartment volume during sleep compared to resting wakefulness albeit no ADC changes in the cortex [27]. To increase the sensitivity of the diffusion analysis we included ADC captured at low-, mid-, and high b-factors as well as kurtosis. A large number of b-factors ranging from very low (20 s/mm^2^) to high (2518 s/mm^2^) were collected for calculation of ADCs and kurtosis and compared between the two anesthetics. Kurtosis is governed by non-Gaussian diffusion and is sensitive for detecting microstructural states associated with ISF volume reduction such as cytotoxic edema and glioblastoma both of which elevated kurtosis [72, 73]. Although we implemented a sensitive diffusion sequence, we were not able to pick up differences between the two anesthetic regimens (Tables 2, 3).

It is possible that the parenchymal enlargement with ISO compared to DEXM-I represents minute differences in blood volume or blood flow which might not be detectable by diffusion imaging. However, if this was the case, we would have expected differences between DEXM-I and ISO observed by the T2* maps of the brain reflective of local magnetic susceptibility sensitive to hemodynamic state within the brain [34, 74, 75] to be ‘focal’ rather than global (Fig. 4). Indeed, the increases of T2* values in the ISO group were widespread and included cortical as well as subcortical regions (Fig. 4). ISO is known to increase cerebral blood flow (CBF), decrease the cerebral metabolic rate of glucose and the oxygen extraction fraction [76–78], but increase the oxygen concentration in the brain tissue when compared to wakefulness [79]. These cerebral hemodynamic and tissue oxygen signatures with ISO are also associated with characteristic EEG patterns including burst firing, and increased alpha and beta power when compared to those observed with DEXM [80]. Thus, T2* increases in the ISO group in comparison to DEXM-I may reflect these underlying differences. Finally, we also documented difference in heart rate (Table 1). DEXM is known to cause bradycardia and the heart rate was reduced by ~ 20% in comparison to ISO (Table 1). Although increased pulsatility has been shown to augment glymphatic solute and fluid influx[2], DEXM slows down pulsatility. However, DEXM might have induced changes in vasomotion. Vaso-motor movement, which is driven by the contractibility of arterial/arteriolar smooth muscle tone oscillating at the frequency below 0.1 Hz, contribute to the water movement and it has been suggested to be driving force in clearance of toxic wastes [21]. However, more studies are needed in studying contribution of vasomotor action to solute transport.

### Clinical implications

The findings of our study have important clinical implications as well. In today’s clinical anesthesia practice the most common strategy for general anesthesia for surgery is a so-called balanced anesthesia [81]. With a balanced general anesthesia, a combination of different agents are used to produce the necessary states of unconsciousness, amnesia, analgesia, and immobility. Typically, maintenance of the anesthetic state is carried out with an inhalational agent (or propofol) in combination with DEXM and opioids [81]. The concentration of the inhalational agent will vary dependent on the type of surgery and patient (e.g. age, morbidity) and higher or lower concentrations of an inhalational agent are used in the setting of a balanced general anesthesia. Currently, it is rare to administer inhalational anesthetics alone except for shorter, non-invasive cases (e.g. MRI diagnostics [82]), or for less invasive procedures in pediatric patients. Our study showed that the higher concentration of the inhalational agent perturbed CSF and parenchyma volume conditions when compared with the balanced anesthetic regimen (DEXM-I). With ISO only the CSF compartment volume was reduced, and parenchymal volume slightly increased compared to DEXM-I which will negatively impact glymphatic solute transport. The ISO scenario with reduced CSF and increased parenchymal volume may be disadvantageous and potentially concerning especially in patients who are already at a higher risk of post-operative delirium [83–85]. Thus, lowering the dose of the inhalational anesthetic and/or using supplemental DEXM, especially during longer surgical/anesthesia procedures, may prove beneficial in alleviating post-operative delirium by opening up the CSF pathways and “flushing” out waste in the brain. Translational studies in humans should be carried in the future out to address this hypothesis.

## Conclusions

In this study, we quantitively assessed morphological, diffusion, and T2* changes across two anesthetic regimens which are associated with 2-fold differences in glymphatic transport. We demonstrated CSF volume expansion with DEXM-I (in comparison to ISO) and parenchymal (GM) expansion with ISO (in comparison to DEXM-I), which may explain the differences in glymphatic transport. The fact that ISO (in comparison to DEXM-I) causes parenchymal volume augmentation may be an unwanted biophysical state for CSF and solute flux and should be investigated in the human brain. Clearly, a better understanding of the mechanisms that lead to more vigorous glymphatic transport during anesthesia and peri-operatively is an important step towards improved care for elderly patients with high risk of cognitive impairment and delirium.

## Supplementary Information

**Additional file 1**: **Figure S1.** Diffusion-weighted MRI (DWI) signals obtained from a DMSO phantom at 20 °C (blue) and in rat cortex (red) are plotted as a function of b-factors. The solid blue line represents the mono-exponential fit of the DMSO signal (derived ADC = 680 μm^2^/sec). A: b-factor range (20~2500 s/mm^2^); B: low b-factor range (20-350 s/mm^2^); C: mid b-factor range (350-1100 s/mm^2^ ) and D: high b-factor range (1200-2500 s/mm^2^).

**Additional file 2**: **Figure S2**. Simulated DWI signals (dots) are plotted as a function of b-factors under different kurtosis using the parameters derived from a previous study by Iima et al.1. kurtosis was reduced from the reference value of 1.0 (red) to 0.8 (blue) and 0.6 (black). Solid lines represent mono-exponential fit only within the high b-factor range for each of the three kurtosis values.

**Additional file 3**: Additional reference.

## Data Availability

The datasets analyzed for the current study are available from the corresponding author on reasonable request. All data generated or analyzed are included in the manuscript.

## Abbreviations

Aβ: Amyloid beta
AD: Alzheimer’s disease
ADC: Apparent diffusion coefficient
CNS: Central nervous system
CSF: Cerebrospinal fluid
DEXM-I: Dexmedetomidine supplemented with low-dose isoflurane
DMSO: Dimethyl sulfoxide
DWI: Diffusion weighted MRI
FA: Flip angle
FDR: False discovery rate
GM: Grey matter
ISF: Interstitial fluid
ISO: Isoflurane
K: Kurtosis
LSD: Least significant difference
MGE: Multigradient echo scan
MRI: Magnetic resonance imaging
NA: Signal averages
NE: Norepinephrine
PDW: Proton density weighted
KX: Ketamine/xylazine
RARE: Rapid acquisition with relaxation enhancement
ROI: Region of interest
TE: Echo time
TIV: Total intracranial volume
TR: Repetition time
VBM: Voxel-based morphometry
WM: White matter
