# Supplementary figures and images for: Disparate volumetric fluid shifts across cerebral tissue compartments with two different anesthetics
Source: Fluids Barriers CNS. 2021 Jan 6;18:1. doi: 10.1186/s12987-020-00236-x (PMC7788828; doi:10.1186/s12987-020-00236-x)

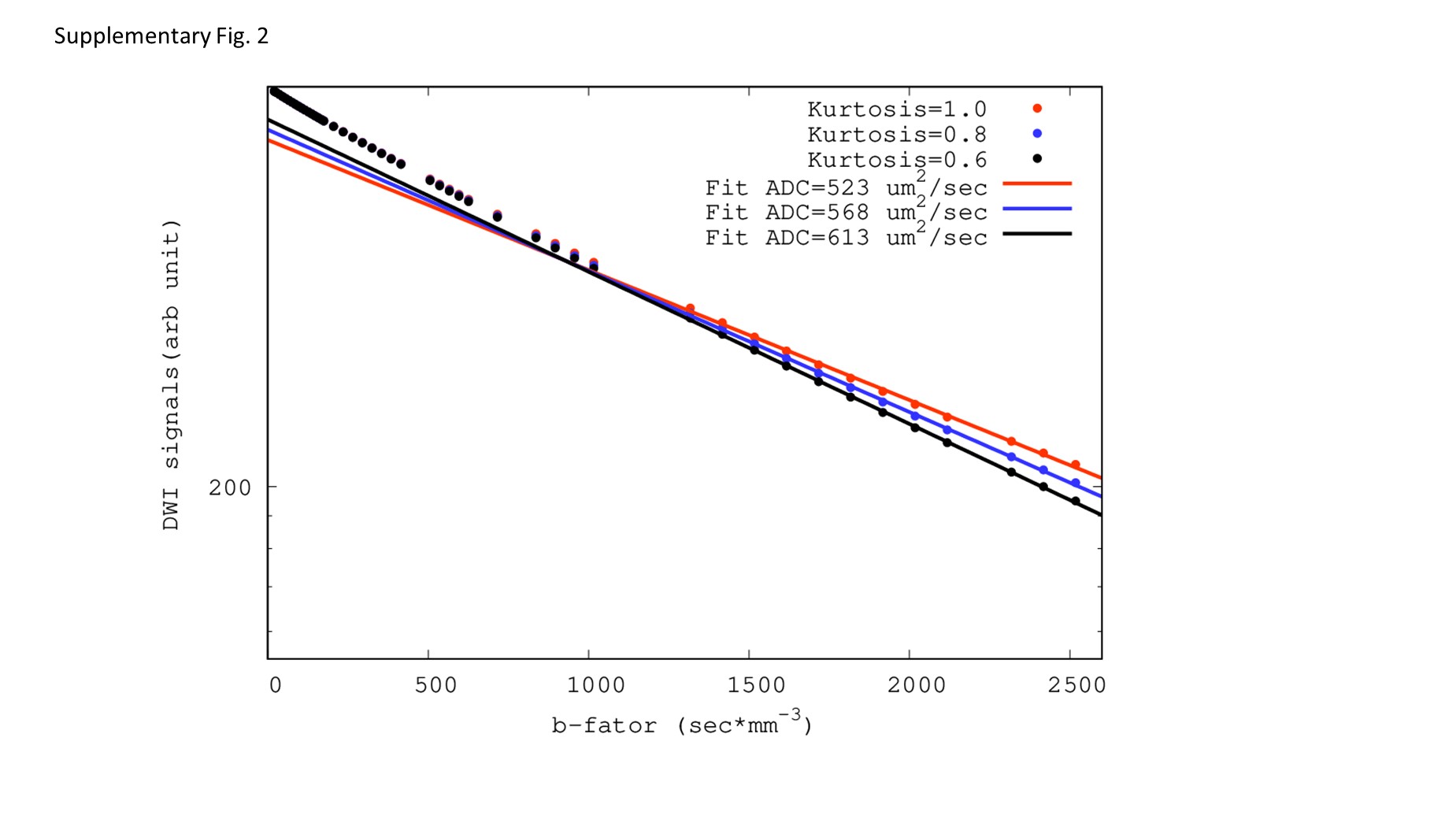

Supplement: Supplementary file 1 — Additional file 1: Figure S1. Diffusion-weighted MRI (DWI) signals obtained from a DMSO phantom at 20 °C (blue) and in rat cortex (red) are plotted as a function of b-factors. The solid blue line represents the mono-exponential fit of the DMSO signal (derived ADC = 680 μm2/sec). A: b-factor range (20~2500 s/mm2); B: low b-factor range (20-350 s/mm2); C: mid b-factor range (350-1100 s/mm2 ) and D: high b-factor range (1200-2500 s/mm2). [file 12987_2020_236_MOESM1_ESM.jpg]

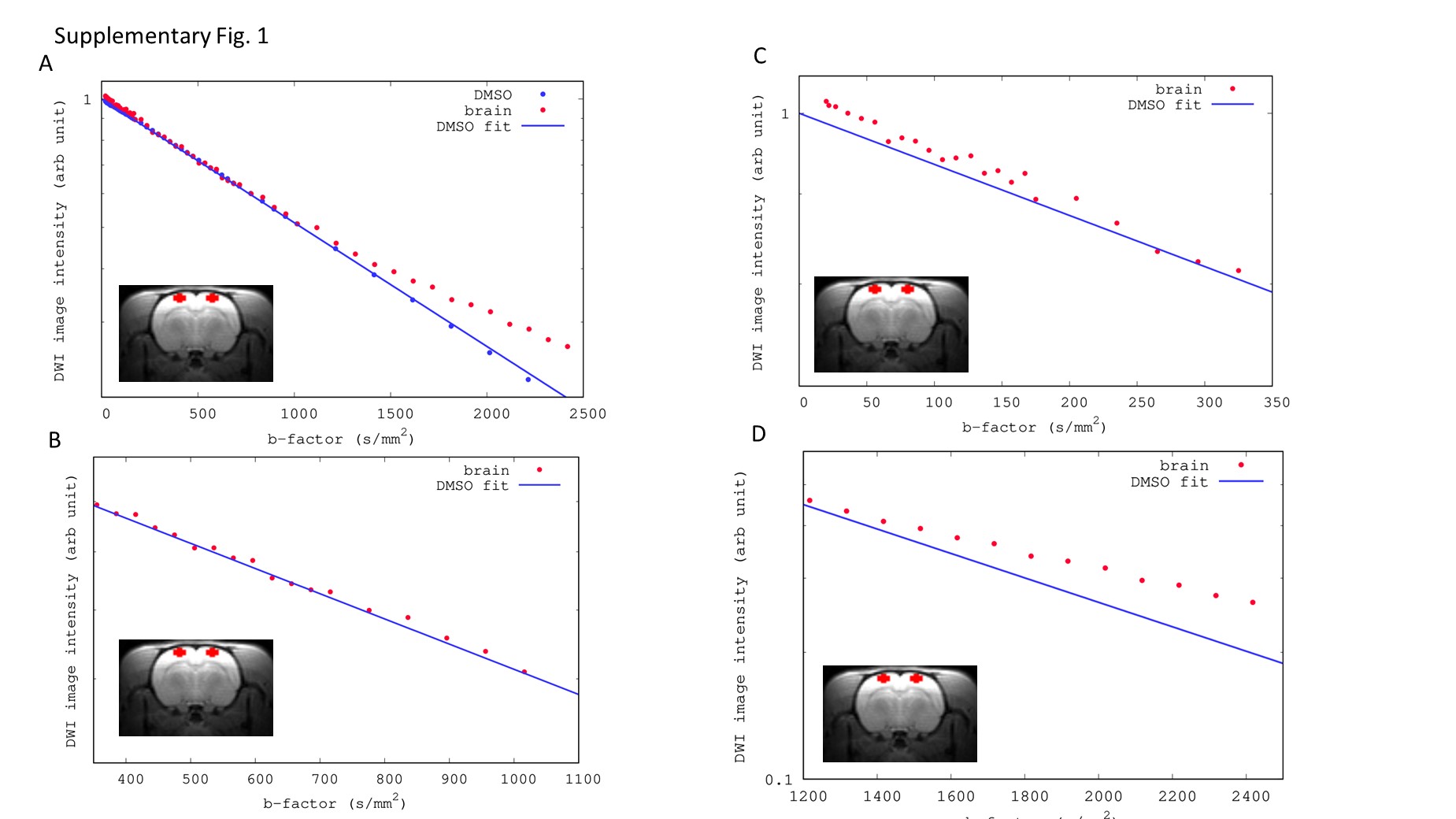

Supplement: Supplementary file 2 — Additional file 2: Figure S2. Simulated DWI signals (dots) are plotted as a function of b-factors under different kurtosis using the parameters derived from a previous study by Iima et al.1. kurtosis was reduced from the reference value of 1.0 (red) to 0.8 (blue) and 0.6 (black). Solid lines represent mono-exponential fit only within the high b-factor range for each of the three kurtosis values. [file 12987_2020_236_MOESM2_ESM.jpg]
