# Supplementary material for: Disparate volumetric fluid shifts across cerebral tissue compartments with two different anesthetics
Source: Fluids Barriers CNS. 2021 Jan 6;18:1. doi: 10.1186/s12987-020-00236-x (PMC7788828; doi:10.1186/s12987-020-00236-x)
Supplement: Supplementary file 3 — Additional file 3: Additional reference. [file 12987_2020_236_MOESM3_ESM.docx]

**Additional File 3: Additional reference**

1. Iima M, Reynaud O, Tsurugizawa T, Ciobanu L, Li JR, Geffroy F, Djemai B, Umehana M, Le Bihan D: Characterization of glioma microcirculation and tissue features using intravoxel incoherent motion magnetic resonance imaging in a rat brain model. Invest Radiol 2014; 49: 485-90
